# A Methodological Framework for Assessing Social Presence in Music Interactions in Virtual Reality

**DOI:** 10.3389/fpsyg.2021.663725

**Published:** 2021-06-11

**Authors:** Bavo Van Kerrebroeck, Giusy Caruso, Pieter-Jan Maes

**Affiliations:** Department of Art, Music, and Theatre Sciences, IPEM, Ghent University, Ghent, Belgium

**Keywords:** presence, virtual reality, music, embodiment, social interaction, interpersonal coordination, ecologically valid research

## Abstract

Virtual reality (VR) brings radical new possibilities to the empirical study of social music cognition and interaction. In the present article, we consider the role of VR as a research tool, based on its potential to create a sense of “social presence”: the illusory feeling of being, and socially interacting, inside a virtual environment. This makes VR promising for bridging ecological validity (“research in the wild”) and experimental control (“research in the lab”) in empirical music research. A critical assumption however is the actual ability of VR to simulate real-life social interactions, either *via* human-embodied avatars or computer-controlled agents. The mediation of social musical interactions *via* VR is particularly challenging due to their embodied, complex, and emotionally delicate nature. In this article, we introduce a methodological framework to operationalize social presence by a combination of factors across interrelated layers, relating to the performance output, embodied co-regulation, and subjective experiences. This framework provides the basis for the proposal of a pragmatic approach to determine the level of social presence in virtual musical interactions, by comparing the outcomes across the multiple layers with the outcomes of corresponding real-life musical interactions. We applied and tested this pragmatic approach *via* a case-study of piano duet performances of the piece Piano Phase composed by Steve Reich. This case-study indicated that a piano duet performed in VR, in which the real-time interaction between pianists is mediated by embodied avatars, might lead to a strong feeling of social presence, as reflected in the measures of performance output, embodied co-regulation, and subjective experience. In contrast, although a piano duet in VR between an actual pianist and a computer-controlled agent led to a relatively successful performance output, it was inadequate in terms of both embodied co-regulation and subjective experience.

## Introduction

Virtual reality (VR) encompasses a plethora of technologies to create new environments, or simulate existing ones, *via* computer-generated multisensory displays (Sutherland, 1968; Taylor, 1997; Scarfe and Glennerster, 2019). Complementary to multisensory displays are technologies for capturing physical body movement to facilitate embodied control and interactions with(in) computer-generated (virtual) environments (Yang et al., 2019). VR technologies provide hence a technological mediation between performed actions and multisensory perceptions, extending the natural sensorimotor capacities of humans into the digital world (Kornelsen, 1991; Biocca and Delaney, 1995; Ijsselsteijn and Riva, 2003). Crucially however, VR typically aims at making its mediation invisible, creating for users the illusory feeling of nonmediation; a feeling coined with the concept of “presence” (Lombard and Ditton, 1997; Riva, 2006). This concept of presence may encompass multiple categories, related to the physical environment, the user’s own body, as well as its social environment. The first category – physical presence or telepresence – pertains to the illusory feeling for users of actually being present in another environment than the one they are physically in (Minksy, 1980; Sheridan, 1992; Slater and Sanchez-Vives, 2016). Another category, self-presence is rooted in the capacity of VR to map the physical body movements of a user onto the moving body of a virtual avatar. The potential of embodying virtual avatars allows to create the illusory feeling for a user of owning, controlling, and being inside another body than its physical one (self-presence) (Kilteni et al., 2012; De Oliveira et al., 2016; Braun et al., 2018; Matamala-Gomez et al., 2020). In addition, (bodily) acting within a virtual social context may create a sense of being together (co-presence), or interacting with others (social presence) while actually being physically remote (Short et al., 1976; Garau et al., 2005; Parsons et al., 2017; Oh et al., 2018).

In the current paper, we advocate for establishing VR as a research tool for studying social music interaction and sense-making. We see the relevance of VR precisely within its capacity to create a sense of presence across the different categories described above. In the first part of the article, we will discuss in more detail how a VR-based approach has its roots in earlier, human-centered research across a broad range of scientific disciplines and how it holds potential for empirical research on social music cognition and interaction.

In the remainder of the article, we focus on a fundamental prerequisite for establishing the advocated VR-based research method; namely, the idea that VR can actually create a sense of social presence. This is particularly challenging given that music provides a highly particular context of human social interaction. It involves the body as a source of expressive and intentional communication between co-performers, carried out through a fine-tuned and skillful co-regulation of bodily articulations (Leman, 2008; Leman and Maes, 2015). This co-regulation of bodily articulations is a complex process, involving many body parts, and taking place across multiple, hierarchically organized spatial and temporal scales (Eerola et al., 2018; Hilt et al., 2020). Successful co-regulation requires hence that action-relevant information at multiple scales is properly exchanged through the different sensory modalities. In particular, the auditory and visual sense are important in signaling communicative cues, related to music-structural aspects and emotional expression (Williamon and Davidson, 2002; Goebl and Palmer, 2009; Keller and Appel, 2010; Coorevits et al., 2020). This complex, embodied nature of music interaction puts considerable demands to communication technologies that aim at mediating social music interactions *via* digital ways. VR however is, in principle, highly promising as it allows to animate full-body, three-dimensional virtual humans based on real-time mapping of body movements of actual people captured by motion capture systems (avatars) or based on computer-modeling and simulation of human behavior (agents) (Cipresso, 2015). These animated, three-dimensional avatars and agents can be observed by others from a freely chosen and dynamic first-person perspective providing a foundation for the complex information exchanges required for successful music interactions. This turns a VR environment into a potential digital meeting space where people located in physically distinct places, together with computer-generated virtual humans, can interact musically with one another. However, it is crucial to further assess the quality of social interactions with virtual avatars and agents (Kothgassner et al., 2017) and to assure the required levels of realism and social presence.

For that purpose, a main objective of this article is to introduce a methodological framework to assess social presence in virtual music interactions. We thereby consider social presence as a multi-layered concept rooted in, and emerging from, the behavioral and experiential dynamics of music interactions. We are able to assess these dynamics by integrating direct data measurement related to performance output, body movement, and (neuro)physiological activity with subjective self-report measures. As such, the framework facilitates the design of interactions, avatars, and agents to obtain empirical data and investigate aspects of the subjective experience such as for example empathy, intimacy, and togetherness. In the second part of the paper, we apply the framework to a case-study of a social music interaction in VR. The case-study presents real and virtual interactions between two expert pianists, a pianist with an avatar and a pianist with an agent and demonstrates similarities and differences revealed throughout the framework’s layers. Finally, we conclude the paper with a discussion on the relevance of our framework using insights from the case-study’s analysis and present directions for future work.

## VR: A Research Tool for Studying Social Interactions

Around the 21st century, VR started to develop as a valuable methodological tool in human-centered research, including the social and cognitive (neuro)sciences (Biocca, 1992; Biocca and Delaney, 1995; Blascovich et al., 2002; Fox et al., 2009; Parsons, 2015; Slater and Sanchez-Vives, 2016; Parsons et al., 2017; Pan and de Hamilton, 2018), philosophy (Metzinger, 2018), the humanities (Cruz-Neira, 2003), product design (Berg and Vance, 2017), marketing (Alcañiz et al., 2019), medicine (Riva et al., 2019), and healthcare (Teo et al., 2016). Although VR-based research exhibits a richness in variety and discipline domains, the relevance of VR in human-centered research can, in general terms, be captured by two specific traits; namely the ability of VR to simulate existing, “real-life” contexts (simulation trait) and its ability to extend human functions or to create new environments and contexts (extension trait).

The simulation trait of VR relates to the inherent paradox in traditional approaches in empirical research. To obtain valid results and insights, the researcher is motivated to observe phenomena “in the wild” without interventions. However, this approach allows little control over stimuli; often has to cope with a number of confounding variables and provides challenges to perform reliable measurements. On the other end, the researcher performs experiments in a controlled lab setting to obtain generalizable results. This approach however is often overly reductionistic and not ecologically valid. The use of VR technology allows to bridge these extremes by simulating real-life settings in a controlled environment. In that sense, VR holds the potential of bringing the external validity (“research in the wild”) and internal validity (“research in the lab”) of social (music) cognition research closer together (Parsons, 2015; Kothgassner and Felnhofer, 2020). It can be understood as an alternative empirical research paradigm (Blascovich et al., 2002), offering substantial additional benefits over traditional research practices in laboratory or field conditions. The use of VR allows precise control over multimodal, dynamic, and context rich stimuli (Parsons et al., 2017) while retaining a level of realism required for realistic responses. Despite the need for technological expertise, research practices using VR are becoming more accessible and standardized and can thus provide representative sampling and better replicability (Blascovich et al., 2002). Given the digital nature of creating VR contexts and the requirement of appropriate sensorimotor sensors, VR technology also offers flexibility in the means of and choices in recording data.

A second trait can be related to McLuhan (1964) understanding of technology as an extension of the human body, mind, and biological functions. This view resonated in the early accounts of VR pointing to the ability of VR to create sensorimotor and social experiences not possible or desirable in the actual physical world. Accordingly, VR was defined in terms of a “medium for the extension of body and mind” (Biocca and Delaney, 1995), creating “realities within realities” (Heim, 1994) or “shared/consensual hallucinations” (Gibson, 1984; Lanier, 1988) “bounded […] only by desire and imagination” (Benedikt, 1991). Important to note is that, in most of current human-centered research, this ability of VR is seldomly employed as a form of mere escapism from the physical world. In contrast, VR is mostly used to “make us intensely aware of what it is to be human in the physical world, which we take for granted now because we are so immersed in it” (Lanier, 1988). Accordingly, the use of “impossible stimuli” and illusions generated in VR have contributed substantially to a better understanding of profound aspects of human embodied cognition and social interaction (Parsons et al., 2017; Metzinger, 2018). For instance, VR technology is capable of selectively modulating our perception of space (Glennerster et al., 2006), time (Friedman et al., 2014), (social) cognition (Tarr et al., 2018), and the body (Petkova and Ehrsson, 2008). It has the potential to influence different representational layers of the human self-model (Metzinger, 2018) leading to phenomena such as virtual embodiment, (virtual) body swapping (Petkova and Ehrsson, 2008; De Oliveira et al., 2016) and increasingly frequent and complex “social hallucinations” (Metzinger, 2018).

Given these traits and their potential, the use of VR in music research has increased over the recent decade (Çamci and Hamilton, 2020). A first category of studies primarily leveraged the simulation trait of VR. They created real-life virtual settings in which to investigate various topics, such as music therapy (Orman, 2004; Bissonnette et al., 2016), music education (Orman et al., 2017; Serafin et al., 2017), music performance (Williamon et al., 2014; Glowinski et al., 2015), and the relation between sound and presence (Västfjäll, 2003; Kern and Ellermeier, 2020; Kobayashi et al., 2020). A good example of simulating a real-life setting is given by Glowinski et al. (2015), who investigated the influence of social context on performance. Specifically, Glowinski and colleagues asked participants to perform a musical task in a virtual concert hall while controlling for audience gaze. Other studies focused more on extending real-life contexts. They range from the search toward new virtual instruments (Honigman et al., 2013; Berthaut et al., 2014; Serafin et al., 2016; Hamilton, 2019) to new interactions and the development of interaction design principles (Deacon et al., 2016; Atherton and Wang, 2020). While we made a clear conceptual distinction between the simulation and extension trait, this distinction is opaquer in practice. An effective research paradigm has been to simulate a musical scenario in VR, subsequently extending some human function such as modulating the feeling of body ownership using virtual embodiment, to investigate behavioral changes (Kilteni et al., 2013).

A critical requirement however for using VR as a research tool in the study of social music interaction is the ability to establish social presence: the illusory feeling of actually being together and interacting meaningfully with human-embodied avatars or computer-controlled agents in VR. Research on social presence may contribute to social music cognition and interaction in two important ways. First of all, referencing again to the quote by Lanier (1988), social music interaction in VR forces researchers to think about, and develop knowledge on, the general nature of human social cognition and sense-making, “which we take for granted now because we are so immersed in it.” Secondly, under the condition that social presence can be reliably established, it becomes possible to accurately control and manipulate the many variables that characterize a music interaction, including the context in which the interaction occurs. For instance, it becomes possible to control the perspective that people have on one another, the distance at which they are positioned, the sensory coupling between people, the appearance of people (for example, facial expression, age, and gender), environmental properties, the actual musical behavior, and bodily performance of VR agents (for example timing, quantity of motion), among other variables. This offers almost limitless possibilities to extend the empirical investigation of the principles of social music interaction and sense-making within (simulated) ecologically valid music environments. In the following section, we describe the methodological framework that we propose to define, measure, and test social presence in VR music interaction contexts.

## A Methodological Framework to Assess Social Presence in VR

Most research so far has relied on self-report questionnaires to assess the subjective feeling of social presence (Cui, 2013; Oh et al., 2018). The mere use of subjective ratings however poses important limitations, as these provide only indirect and *post hoc* measures of presence, lack subtlety and are often unstable and biased (Cui, 2013). In the current article, we propose an alternative, pragmatic approach, considering social presence as emerging from the performative, behavioral, and experiential dynamics inherent to the social interaction. This allows the assessment of social presence using a combination of qualitative, performer-informed methods, and quantitative measures of the performance, behavior, and (neuro)physiological responses of users by operationalizing them into concrete, direct, and measurable variables. Crucially, in this pragmatic approach, we define the level of social presence as the extent to which social behavior and responses in simulated VR contexts resemble behavior and responses in corresponding real-life musical contexts (Minksy, 1980; Slater et al., 2009; Johanson, 2015; Scarfe and Glennerster, 2019).

**Figure 1:**
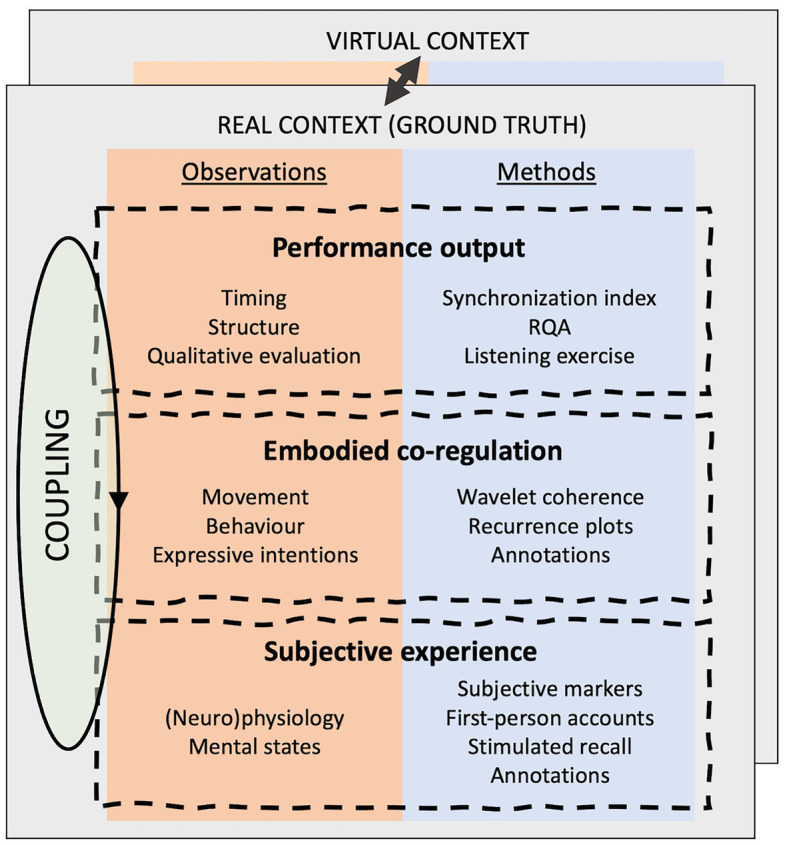
Overview of the methodological framework to operationalize social presence in virtual reality (VR) music contexts. The core of the framework consists of a comparative analysis of a simulated virtual context, with the corresponding real-life music context (which functions as “ground truth”) across three interrelated layers; performance output, embodied co-regulation, and subjective experience. (RQA = Recurrence quantification analysis)

To allow comparison between virtual and real-life scenarios, we rooted our framework for social presence in the “interaction theory,” which currently is the most dominant theory in the social sciences to understand social cognition and sense-making (Gallagher, 2001; De Jaegher and Di Paolo, 2007; Kiverstein, 2011; Froese and Fuchs, 2012; Gallotti and Frith, 2013; Schilbach et al., 2013; Fogel, 2017). Proponents of the interaction theory consider social cognition essentially as an embodied and participatory practice, emerging in real-time co-regulated interaction and not reducible to individual processes. In line with this account, we consider successful co-regulation as a foundational criterion to establish social presence in VR. Importantly, in our framework, we consider social co-regulation both from the viewpoint of the quantifiable bodily and multisensory patterns of interpersonal interaction, as from the viewpoint of the intersubjective experience and participatory sense-making (De Jaegher and Di Paolo, 2007). Together with the actual musical outcome, these two interrelated aspects of social co-regulation form the three main layers of our framework to determine the degree of social presence in VR music contexts. Layers of the framework are shown in Figure 1.

### Layer 1: Performance Output

The performance output layer relates to the (un/successful) realization of musical ideas or goals, which may be strictly prescribed in musical scores, loosely agreed upon, or emerge in the performance act itself, depending on the performance type and context. Music performance analysis has been advanced by research and development in the domain of music information retrieval, providing ample techniques and methods for assessing music performance properties (Lerch et al., 2020). These are typically extracted from audio recordings, although other multimodal signals such as body movement are increasingly being used. Further, we advocate for taking into account time-varying features related to timing, synchronization, (joint) multiscale recurrence patterns, and complexity measures as these may signal the quality of the performance output. These quantitative measures should ideally be complemented with qualitative, performer-inspired methods to reliably interpret the quantitative outcome measures. They include subjective evaluations in the form of aesthetic judgments of the performance output by the performers themselves.

### Layer 2: Embodied Co-regulation

A successful musical output relies on a skillful, joint coordination of co-performers’ actions and sounds. In line with the interaction theory on social cognition described above, we consider social music interaction as a dynamic and continuously unfolding process of co-regulation, in which performers mutually adjust to one another in a complex interplay of action and multimodal perception. This process of co-regulation integrates various levels and mechanisms of control, ranging from low-level spontaneous coordination based on dynamical principles (Kelso, 1995; Tognoli et al., 2020), to higher-level learning, predictive processing, and active inference (Sebanz and Knoblich, 2009; Gallagher and Allen, 2018; Koban et al., 2019). In our proposed methodological framework, we specifically aim at capturing patterns, relationships, and recurrences in the process of co-regulation at the level of the interacting system as a whole. We thereby advocate for the integration of time-series analyses from the domain of dynamical systems theory, as these are ideally suited to unveil dynamic patterns of interpersonal coordination across multiple body parts and temporal and spatial scales (Eerola et al., 2018; Hilt et al., 2020). Patterns can then be found on multiple levels such as in the attention dynamics from a participant’s gaze direction, in expressive gestures with communicative cues from head nodding as well as in the structures of full-body movements resulting from body sway synchronization. The ability to quantify bodily and multisensory patterns of co-regulated interaction between music performers in VR-mediated music contexts is foundational in our approach, as in our view, successful co-regulation is a decisive factor in performers’ feelings of social presence.

### Layer 3: Subjective Experience

This layer deals with the subjectively experienced interaction qualities and sense-making processes of individuals. It contains a combination of quantitative and qualitative methods to link mental states, (expressive) intentions and meaning attributions to observations in other layers. The quantitative methods include the analysis of (neuro)physiological signals as they can give access to low-dimensional aspects of the conscious experience. For instance, electromyography (Ekman, 1992) and pupillometry (Laeng et al., 2012) among others, have proven to provide valid markers of cognitive and affective user states in virtual performance contexts, such as attention and workload measures, vigilance, affect, and flow (Schmidt et al., 2019). Heart-rate and skin-conductance (Meehan et al., 2002), as well as electroencephalography (Baumgartner et al., 2006) represent good candidates as they are capable of directly assessing the feeling of presence in virtual performance contexts. Complementary, from a more qualitative and performer-oriented point of view, one can ask participants for time-varying ratings of their intentional (joint-)actions and expressions through audio-video stimulated recall (Caruso et al., 2016). In addition, *via* self-report questionnaires, one can probe for mental states, such as social presence, flow, and feelings of togetherness (Witmer and Singer, 1998; Lessiter et al., 2001; Martin and Jackson, 2008). Finally, because of the multi-layered nature of social presence, open questions, and semi-structured interviews focused on individual experience of the virtual other can help to fill analysis gaps and interpret quantitative findings across the different layers.

### Operationalization Within a Performance Setting

Layered frameworks have been helpful in earlier research for structuring the investigation into music interactions (Camurri et al., 2001; Leman, 2008). The layered framework here extends these approaches with a focus on the complementary nature of a mixed-method approach and the time-varying aspects, viewing the musical interaction as consisting of multiple interdependent parts. Layers are functionally coupled by non-linear relations allowing the emergence of patterns in time-varying dynamics in each layer. They frame and couple the dynamics of quantitative bodily and multisensory coordination patterns with the (inter)subjectively felt qualities of the music interaction. The framework aims to serve as a template to map this dynamic landscape of time-varying dynamics and aid in the uncovering of insightful states and transitions in a broad range of social interactions. It allows the investigation of social presence in different performance settings and distinguishes interactions using a specific operationalization of qualitative and quantitative methods in each layer. These operationalizations can vary from simple setups with for example, audio-, video-recordings, and annotations in the performance, co-regulation, or subjective layer to the more complex setups that will be presented in the case-study below. One performance setting might have time-delay or phase as variable of interest in the performance layer, while another might focus on frequency. Some settings will require the observation of neurophysiological signals while others might focus on movement data or self-reporting. Direct assessment is preferred over post-experimental reports to avoid the influence of self-referential cognitive processes or interfering in the interaction. Application of the framework then allows to identify interactions with for example close coordination and intense subjective experiences but nevertheless inferior performance such as when two tennis players are struggling to have long rallies but nevertheless experience heightened attention and synchronized movements. Other interactions can have successful performance outcomes, but fail in creating fertile metastable dynamics (Kelso, 1995; Tognoli et al., 2020) in other layers. Examples can be found in the interactions between human users and current state-of-the art artificial intelligence in video games, humanoid robots, or virtual assistants that lack successful (embodied) co-regulation and dynamic intentional relations.

## A Case-Study: Piano Phase

“Piano Phase” (1967) is a composition for two pianos, written by minimalist composer Steve Reich. The piece applies his phase-shifting technique as structuring principle of the composition and is written for two pianists. The piece was chosen as case-study, as it provides an excellent musical case to assess social presence in VR music performance across the different layers in our proposed methodological framework. First, the performance output, the instructed phase shifts throughout time, can be objectively assessed and compared across different performances. Second, the performance requires skilled co-regulation between pianists in order to successfully perform. And third, as also Reich acknowledges, the performance of the piece has profound psychological aspects, related to sensuous-intellectual engagement, and strong (inter)subjective experiences of heightened attention, absorption, and even ecstasy.

### Research Question

The case-study was meant to empirically evaluate and test our pragmatic approach for the definition and measurement of social presence in real-time VR music performances based on the proposed methodological framework. For that purpose, we designed different performance contexts that enabled us to compare performances of Piano Phase in VR, with a corresponding (ground truth) performance of Piano Phase under normal, “real-life” conditions (see Design section). In all conditions, we captured an elaborated set of quantitative data related to the experience, behavior, and performance of the pianist duo. In addition, we complemented this data with qualitative methods to integrate experiences and intentions from a performer point of view. Based on this quantitative and qualitative data and guided by the layered analysis model inherent to the proposed methodological framework, we could then conduct comparative analyses across the performance contexts to evaluate social presence the test subject experienced in VR.

### Participants

The case-study involved three expert pianists: one test subject and two research confederates. The experimental protocol was reviewed and approved by the ethical commission of the University of Ghent. All pianists had over 10 years of professional music experience. The test subject (female, 38 years, in the following termed “Test pianist”) was not familiar with the piece Piano Phase through earlier performances and did not have earlier VR experiences. A second pianist (male, 32 years, in the following termed “Confederate pianist 1”) functioned as research confederate in the first two conditions and had concert experiences in performing Piano Phase. Finally, the third pianist (female, 44 years, in the following termed “Confederate pianist 2”) was another research confederate in the third condition and co-author of this study. She had no experience in performing the piece but did have experience with VR.

### Design

**Figure 2:**
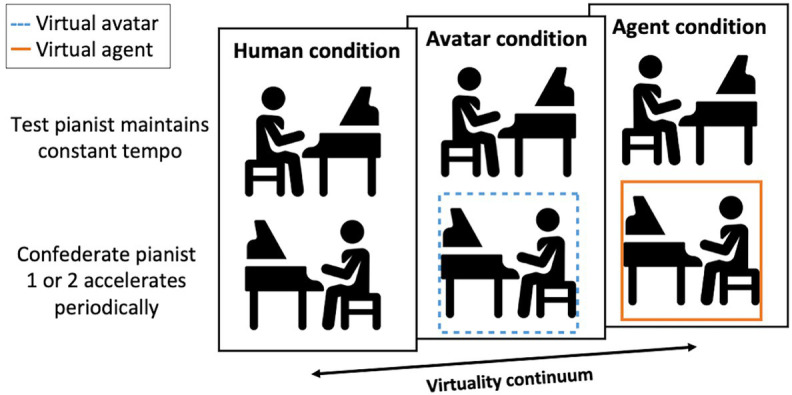
Conditions in the case-study (Confederate pianist 1 plays in the Human and Avatar condition, a recording of Confederate pianist 2 plays in the Agent condition).

The experiment consisted of three conditions as presented in the schematic overview in Figure 2. Conditions are placed along a virtuality continuum represented by the arrow in the figure (Milgram et al., 1995) and correspond from left to right to an unmodelled, partially modeled, and fully modeled world. In each condition, the Test pianist performed Piano Phase together with a research confederate while wearing a Head-Mounted-Display (HMD) (Confederate pianist 1 in the first two conditions and Confederate pianist 2 in the third condition). The fundamental distinction between the three conditions was the level of behavioral realism of the confederate partner as perceived by the Test pianist:

1. Human condition (ground truth): the Test pianist and Confederate pianist 1 performed Piano Phase under normal, “real-life” concert conditions. The Test pianist visually perceived Confederate pianist 1 in a natural, physical manner. To match the two other performance conditions, we asked the Test pianist to wear a HMD with the pass-through camera activated. This was done in order to avoid that the constraints of the HMD would function as a confounding factor while maintaining a normal, physical exchange of auditory and visual information between the Test pianist and Confederate pianist.
2. Avatar condition: the Test pianist and Confederate pianist 1 performed together in real-time, but the Test pianist visually perceived Confederate pianist 1 as a human-embodied virtual avatar. The Test pianist was disconnected from visual information coming from the physical environment, and all visual information related to the virtual room, piano keyboard, hands, and the co-performing Confederate pianist 1 was provided to the Test pianist *via* the HMD. Full body movements and musical instrument digital interface (MIDI) piano performance of the virtual avatar were streamed in real-time from the performance of Confederate pianist 1.
3. Agent condition: the Test pianist performed together with a computer-controlled virtual agent. The Test pianist was disconnected from all direct visual information as similar to the Avatar condition. Full body movements and MIDI piano performance of the virtual agent were rooted in pre-recorded time-series data of an actual pianist (Confederate pianist 2), who was asked to perform the same role as the one of the virtual agent (see below, Task). For the virtual agent animation, we used the Kuramoto model to automatically phase-align these pre-recorded time-series data and the accompanying audiovisual VR animation to the real-time performance of the Test pianist. This allowed to accurately control the phase of the musical part of the virtual agent with respect to the Test pianist and hence, to perform the piece dynamically as prescribed by composer Steve Reich. Apart from the virtual agent, all other display factors were similar as in the Avatar condition.

For more information on the display methods, see the Materials and Apparatus section below. We will use Human condition (HC), Avatar condition (AvC), and Agent condition (AgC) abbreviations in the Analysis and Results sections to refer to numerical results from the Human, Avatar, and Agent condition.

### Materials and Apparatus

#### Piano Keyboards

The piano keyboards used for the performances were digital interface MIDI controllers. The Test pianist and Confederate pianist 1 played on a Yamaha P60 and Confederate pianist 2 played on a Roland RD700SX. MIDI information was processed in Ableton Live 10 to generate piano sounds using a Native Instruments Kontakt 6 plugin. Speakers were placed underneath each piano keyboard to assure coherent sound source localization throughout the different performances.

#### Performance Setting and Virtual Simulation Displays

**Figure 3:**
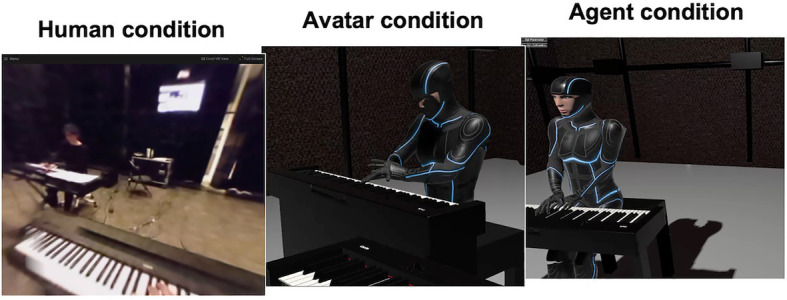
View of the Test pianist in each condition as seen through the Test pianist’s head-mounted display.

The full experiment took place in the Art and Science Interaction Lab of IPEM, a 10 m-by-10 m-by-7 m (height) space surrounded by black curtains that resembles a realistic performance space. The two piano keyboards were placed opposite to each other under an angle of about 60° so pianists could turn toward each other (see Figure 3). The avatar and agent were based on full body movement captures using the Qualisys system described below. Motion capture data were processed in Unity. An important consideration in the study was to also simulate the hands of the Test pianist as earlier research indicated that this may substantially increase the feeling of (self-)presence (Banakou and Slater, 2014). We used the Leap Motion system for that purpose which allowed to track and display fine finger movements.

#### Data Measurement Setup

**Figure 4:**
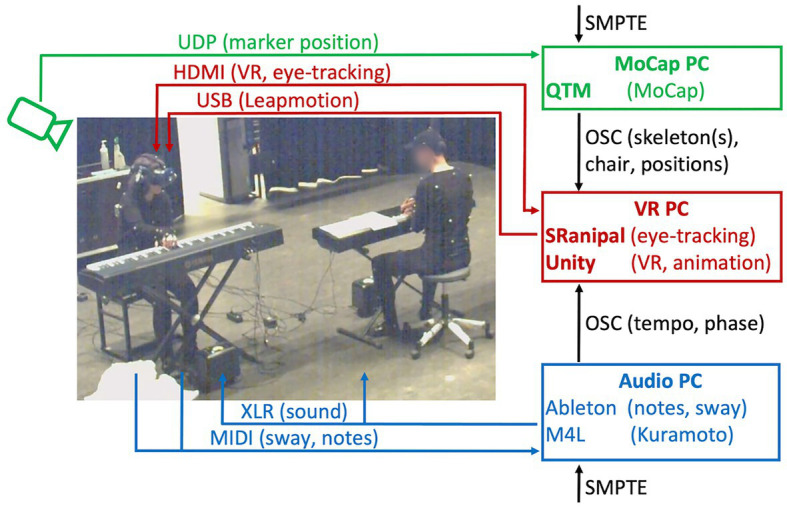
Schematic overview of the case-study’s technical set-up.

Multiple technologies were used to capture and measure bodily behavior and performance aspects of the pianists into quantitative time-series data. Concerning the performance, we recorded MIDI data from the piano keyboards, including MIDI note numbers, note-on/off times, and note velocities. Delays from piano keypresses to audio equaled 16 ± 5 ms. We captured full body movements of all pianists (3D position, 120 Hz) using a multi-camera Qualisys optical motion capture system (OQUS 7+ cameras). Real-time streaming of full-body movement data of Confederate pianist 1 to visualization in the HMD had a latency of 54 ± 11 ms. In addition, video was recorded using a four-camera Qualisys Miqus system. Further, we captured how the Test pianist distributed her body weight on the chair using four pressure sensors mounted underneath the four legs of the piano chair. Finally, we tracked the eye movements of the Test pianist using the Tobii eye-tracking technology from the HMD of model HTC Vive Pro Eye. An overview of the technical set-up is shown in Figure 4.

### Task and Procedure

**Figure 5:**
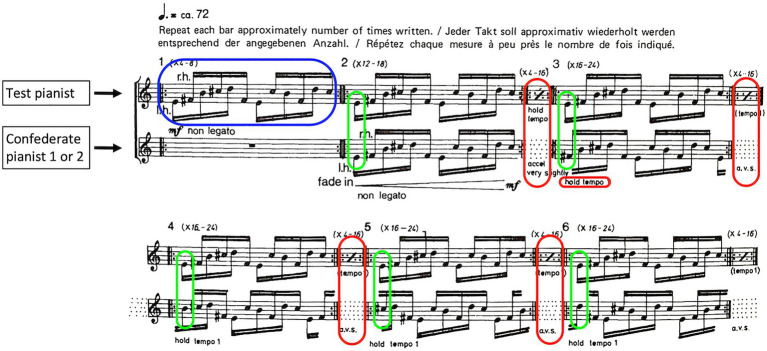
Annotated first 11 bars of Piano Phase (1967) by Steve Reich (blue = melodic pattern, green = phase difference, and red = tempo instruction; reprinted with kind permission by Universal Edition AG, Vienna).

The task for the pianist duos in each condition was to perform Piano Phase as prescribed by the composer Steve Reich. The compositional idea of Piano Phase is to start in unison after which intermittent gradual tempo changes cause increasing phase shifts between the melodic patterns played by each pianist. These shifts, in their dynamic variety of interlocking, then lead to the emergence of a variety of harmonies over the course of the performance until pianists are back in unison. First bars of the piece are shown in Figure 5. Reich’s compositional instruction is as follows: “The first pianist starts at 1 and the second joins him in unison at 2. The second pianist increases his tempo very slightly and begins to move ahead of the first until (say 30–60 s) he is one sixteenth ahead, as shown at 3. The dotted lines indicate this gradual movement of the second pianist and the consequent shift of phase relation between himself and the first pianist. This process is continued with the second pianist gradually becoming an eight (4), a dotted eight (5), a quarter (6), etc., ahead of the first until he finally passes through all 12 relations and comes back into unison at 14 again” (Reich, 2002). In the performance, the pianist that is assigned the top part of the score keeps a constant tempo, while the pianist that is assigned the bottom part performs the gradual phase-shift by gradually increasing his/her tempo. In our study, the Test pianist was always assigned the top part of the score, while Confederate pianists 1 and 2 were assigned the bottom part in the Avatar and Agent condition, respectively. We fixed the number of repetitions for each bar at eight for better experimental control and kept the tempo at 72 BPM (one beat for six 16th notes or a dotted quarter note).

A month before the experiment, the Test pianist was asked to prepare for a performance of the musical composition. The Test pianist received an audio recording of her part with isochronous notes, uniform velocities, and linear accelerations to help with practicing. Upon arrival, the Test pianist was told the experiment consisted of a preparation phase followed by three repetitions of the full piece. She was then given a questionnaire and asked to change into a motion capture suit afterwards. A data skeleton was build using some recordings of her walking and playing the piano after which she was asked to calibrate the HMD’s eye-tracking.

After the explanations, the Test pianist practiced the task with Confederate pianist 1 for about 15 min without wearing the HMD. When both pianists indicated they were ready for the performance, the Test pianist was given the HMD to get accustomed to the virtual environment after which they performed the three conditions. A questionnaire and break were given after each condition. The Test pianist was not told that the agent in the Agent condition was computer-controlled. The experiment concluded with a semi-structured interview about the experience. Five days after the experiment, the Test pianist was asked to listen and evaluate randomized audio recordings of each condition as well as fill in a final questionnaire.

### Analysis

This subsection describes the analysis in the application of the methodological framework on the case-study. It presents the choice of quantitative and qualitative methods used in each layer to obtain insights that will be discussed in the Results section below.

#### Layer 1: Performance Output

In this layer, we investigated if pianists succeeded in executing the compositional instruction. In Piano Phase, both pianists repeat a 12-note pattern of which one pianist accelerates at specific moments for a specific period. This should result in an alternation of stable periods characterized by a consistent relative phase relationship of the note patterns of both pianists and intermittent periods characterized by gradual shifts toward an increased relative phase of the note patterns.

First, we used note onsets of both pianists to determine tempo, inter-onset-intervals (IOIs) and relative phase between pianists. One full phase cycle was defined as 12 notes. Using the relative phase, we calculated the synchronization index (SI) as a measure of stable periods characterized by a consistent relative phase between pianists (Mardia and Jupp, 2009). A SI of 1 represents perfect synchronization and 0 no synchronization.

Next, we looked at musical structure using time-dependent joint Recurrence Quantification Analysis (RQA) of the relative phase. RQA is a non-linear technique for the assessment of dynamic systems and allows to identify transitions and behavior of a system by analyzing patterns of recurrences in a low dimensional phase space from potentially higher dimensional timeseries (Marwan et al., 2007). Since relative phase between pianists represents the driver of the musical composition, RQA on a phase space of relative phase allows to assess transitions and dynamics in the musical performance. RQA metrics, such as the recurrence rate (RR), determinism (DET), and trapping time (TT) were calculated to measure the percentage of recurrences, the percentage of recurrences that are stable and the average length of stable recurrences. We used an embedding dimension of 4 and a time-delay of 0.3 s for a joint RQA. The minimal diagonal length for calculating DET was set to 0.3 s. Joint RQA parameters were obtained by looking at extrema of mutual information and false-nearest neighbor metrics (Marwan et al., 2007). The radius was set to 0.55 to produce around 10% of recurrence across conditions.

Finally, we complemented this quantitative data analysis with subjective evaluations. Five days after the experiment, The Test pianist received six, 15-s, randomized, audio recordings from each condition. She was asked to score each recording on a scale from 0 to 10 on expressiveness (dynamics, accents), timing (rhythm, tempo), interaction quality (collaboration), and appreciation (engaging, positive). In addition, she was asked to leave general remarks for each recording.

#### Layer 2: Embodied Co-regulation

In this layer, we assessed movement, behavior, and (expressive) intentions of the pianists and the means through which pianists actually achieved a successful execution of the musical score. When recording the stimuli for the experiment, pianists used head nods to communicate successful transitions and divided the tasks of counting repetitions and measures among themselves. Communication and co-regulation between pianists played an essential role for a successful performance and realization of the compositional idea behind Piano Phase.

For that purpose, we recorded head movements as 3D spatiotemporal series to obtain communicative cues and signals of mutual understanding in the piano performance (Castellano et al., 2008). To detect correlated frequencies and their phase angles, we performed a wavelet coherence analysis on the series’ main principal component.

We complemented this analysis of dynamics with annotations of specific, expressive gestures in the performance. Concretely, we identified head nods between pianists using the ELAN software (Sloetjes and Wittenburg, 2008) to see whether communicative cues at transitional moments remained consistent across the different performances.

As a measure of coupling and attention toward the other, we recorded the Test pianist’s gaze direction and calculated the angle with the confederate’s head position.

We measured postural sway timeseries using the pressure sensors in the piano chair. We summed these series and used a normalized and unthresholded recurrence plot to look at stable periods and identify transitional moments in the performance (Marwan et al., 2007). The recurrence plot had an embedding dimension of 5 and time-delay of 0.35 s which were defined using mutual information and false-nearest neighbor metrics. Radii were set to produce around 10% recurrences for each condition [(Radius, RR%) equal (0.065, 10.66)_HC_, (0.185, 10.63)_AvC_, and (0.104, 10.55)_AgC_].

#### Layer 3: Subjective Experience

In this layer, we looked at physiological signals and self-reported scores as windows into the Test pianist’s experience during and after the interaction. The immersive tendencies questionnaire (Witmer and Singer, 1998) was taken before the experiment. Self-reported scores were obtained using the flow short scale (Martin and Jackson, 2008), the presence questionnaire (Witmer et al., 2005), and three custom questions about the overall interaction (“Did you enjoy the interaction,” “How close did you feel to your musical partner,” and “How natural did you experience the interaction with your partner”). Additional presence questionnaire items as proposed by Slater and Lessiter were included in the presence questionnaire as well (Witmer et al., 2005). A semi-structured interview about the overall experience was conducted upon conclusion of the experiment. We sent a general question to describe the experience in each condition together with the randomized audio recordings from each condition.

We recorded pupil dilatation using the built-in eye-tracking functionality in the HMD as an estimate of the intensity of mental activity and of changes in attention or arousal (Laeng et al., 2012).

### Results

**Figure 6:**
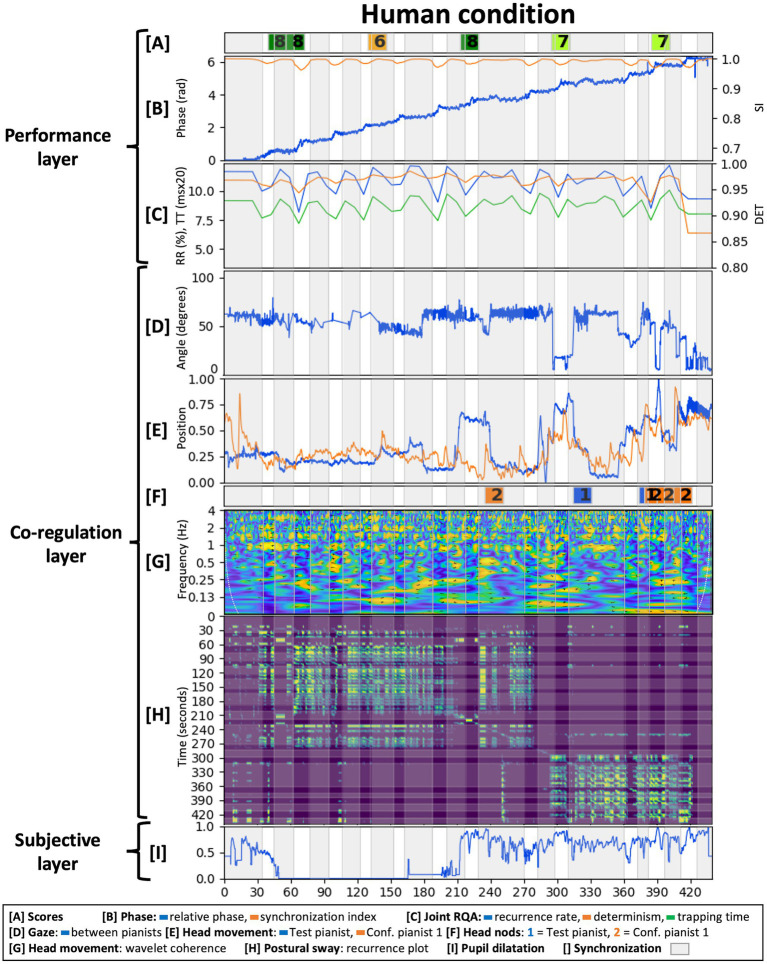
Analysis of the human condition across the three interrelated layers from the proposed methodological framework.

**Figure 7:**
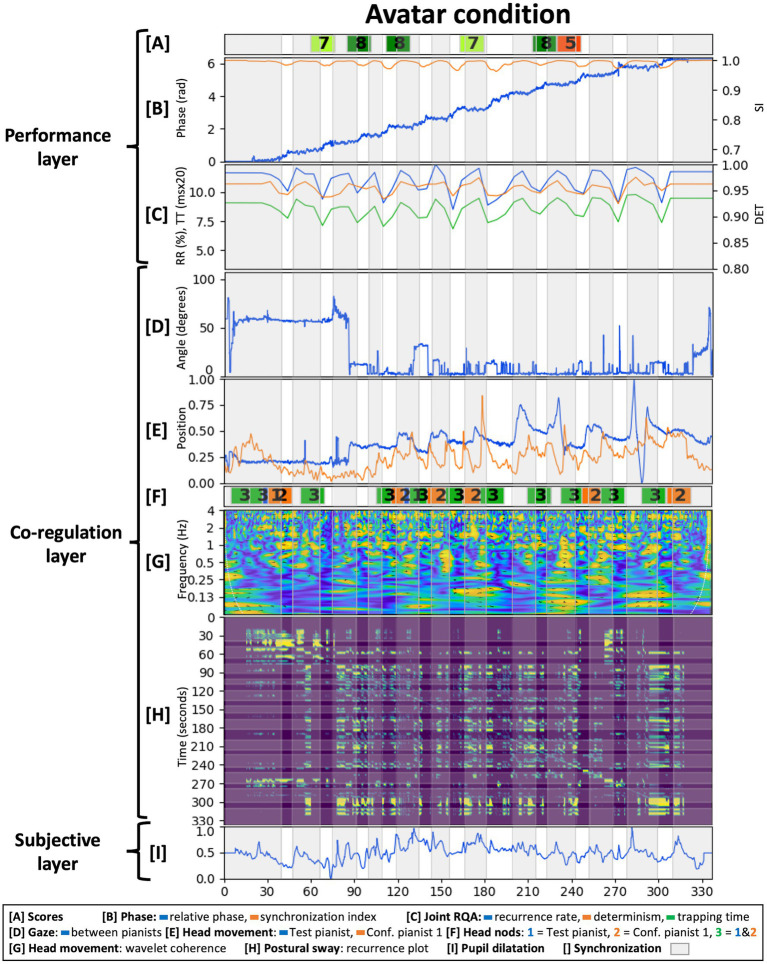
Analysis of the avatar condition across the three interrelated layers from the proposed methodological framework.

**Figure 8:**
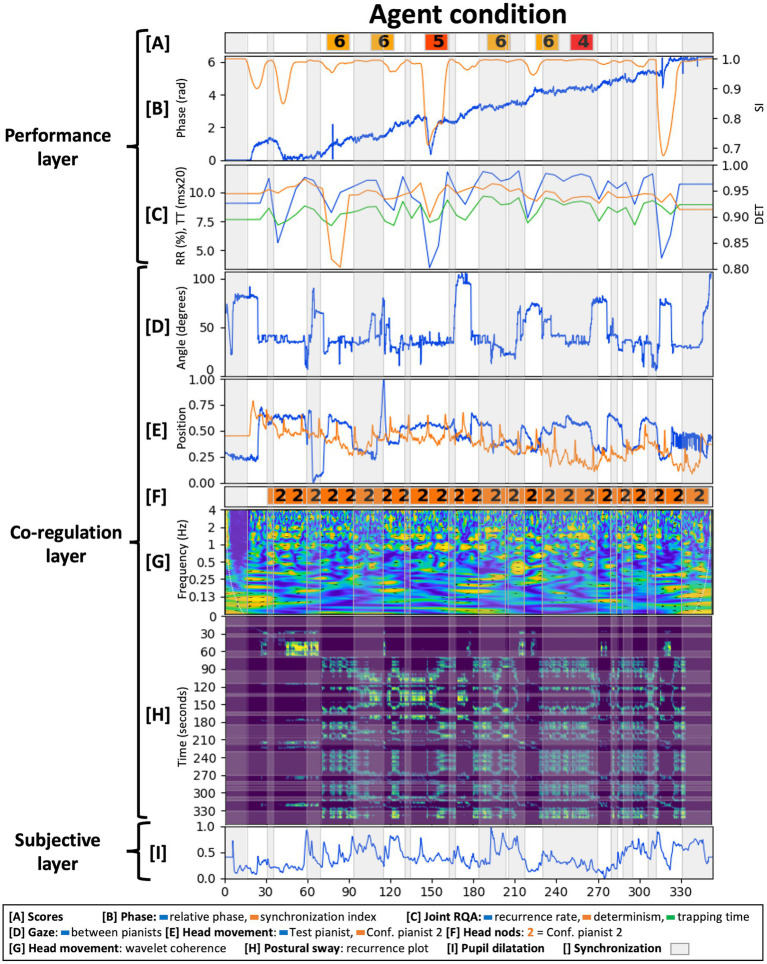
Analysis of the agent condition across the three interrelated layers from the proposed methodological framework.

This section presents the results from analyzing the case-study data. We evaluated each layer of the methodological framework in each condition with results shown in Figures 6–8. Given the fact that only one dyadic couple was observed in all conditions, results are descriptive and meant to provoke reflections leading to the Discussion section below.

#### Layer 1: Performance Output

The score had a tempo indication of 72BPM and participants performed it slightly faster [(mean, STD)_tempo_ equaled (74.73, 4.44)_HC_, (75.90, 4.62)_AvC_, and (74.10, 5.67)_AgC_ BPM]. IOI variability was comparable across conditions with a higher variability for the Agent condition [(mean, STD)_IOIvar_ equaled (7.47, 3.63)_HC_, (7.26, 3.41)_AvC_, and (12.55, 2.73)_AgC_ ms]. As the agent was programmed to be attracted toward 72BPM, the slightly higher tempo of the performance made its tempo corrections larger when accelerating and synchronizing. Across conditions however, the Agent condition’s tempo was closest to the instructed 72BPM.

Bars in the composition represented stable or accelerating tempos with, respectively, constant or shifting relative phase periods in the performance. These periods and their associated transitions were determined by thresholding the synchronization index as indicated by the gray areas in Figures 6–8. We have set the synchronization index threshold at 0.99, which allowed the discrimination of 25 measures in the Human condition. This choice was motivated by the fact that the Human condition was taken as the ground truth and the composition prescribed 25 measures of (de)synchronization. The Agent condition had a more variable bar length distribution with extremes of a long period of synchronization at 240 s and a turbulent moment of successive (de)synchronization around 280 s. Interestingly, a longer period of synchronization is found at the same relative phase in the Human condition as well. This relative phase and resulting harmony might have represented an attractor for the pianists in which it was easy to accelerate in but difficult to accelerate out of.

Overall, increasing relative phase and a fluctuating synchronization index were present across conditions as shown in Figures 6B–8B. The Agent condition did have a turbulent moment in the beginning and the middle of the performance as well as a sudden transition at the end. These moments resulted from the delay in tempo tracking for the virtual agent, an erroneous note in the stimuli and the Test pianist jumping to synchronization with the agent toward the end.

Joint RQA showed alternating periods of (de)synchronization (Figures 6C–8C) and comparable average values for RR, DET, and TT across conditions. The Agent condition did contain more variability, especially during the earlier identified turbulent moments in the performance. The Human condition had a slightly higher average DET value indicating more stable synchronization. Fluctuations in DET values from (de)synchronizing were slightly larger in the Avatar condition compared to the Human condition.

Test pianist’s scores on expressiveness, technical content, and interaction quality of each audio excerpt are indicated in Figures 6A–8A (mean scores: HC = 7.33; AvC = 7.17; AgC = 5.5). The Agent condition received lower scores although differences with the other conditions seem less severe as compared to the self-reported presence scores presented in layer 3 below. Three excerpts from the Agent condition did get the remark “I do not understand the intention of the pianist.”

In conclusion, performances in each condition were executed relatively well with stable tempos, fluctuating synchronization and RQA measures as instructed by the score. Increased DET fluctuations in the Avatar condition were indicative of a good performance given they are indicative of clearer alternating moments of (de)synchronization. Subjective scores by the Test pianist were good for the Human and Avatar conditions and just above average for the Agent condition. Analysis of the performance layer thus showed a good execution of the composition in the Human and Avatar conditions and showed more trouble performing successfully in the Agent condition. Underlying reasons might have resided in the embodied dynamics of coordination and communication that will be discussed in the next layer.

#### Layer 2: Embodied Co-regulation

The normalized, principal component of the head movement timeseries of both pianists is shown in Figures 6E–8E. Wavelet coherence on these timeseries is shown in Figures 6G–8G. These plots show maxima at multiples of half the average tempo of the performance (72BPM or 1.2 Hz) with a less outspoken pattern for the Agent condition. As the score had two beats per measure, it shows how pianists synchronized their head movements coherent with the musical structure. Head movement in the (0.9, 1.5) Hz band had a flat distribution of relative phase angles between pianists across conditions [(mean, resultant vector length) equaled (73°, 0.105)_HC_, (−140°, 0.127)_AvC_, and (82°, 0.070)_AgC_]. Phase shifts in the music might have transferred to head movements and might suggest pianists were mainly keeping time for themselves.

Annotations of head nodding between pianists are shown in Figures 6F–8F. The Human condition contained cues from Confederate pianist 1 toward the end, the Avatar condition contained several synchronized head nods between pianists and the Agent condition showed the absence of communication cues from the Test pianist toward the virtual agent. Synchronized head nodding in the Avatar condition also went together with a closely coupled gaze and regular RQA measures. This close coordination was felt by The Test pianist as she commented after the experiment that she missed the “posture mirroring” of the other pianist in the Agent condition as present in the Avatar condition.

Next, one can see differences between conditions for the gaze angle throughout time, between pianists in Figures 6D–8D. The Human condition had the Test pianist mainly looking forward as the other pianist sat at an angle of 60°. Figure 6D shows a significant decrease of the gaze angle at 300 s in the Human condition just before a longer musical synchronization period. This transition is followed by a head nod of Confederate pianist 1, showing how attention shifted toward the Test pianist. The Avatar condition also had an important transition early in the performance after which the Test pianist kept gaze directed toward the Confederate pianist 1. This attention shift resulted in a closer coordination as illustrated by synchronized head nods. In the Agent condition, pianists demonstrated less co-regulation with a gaze angle between pianists that never reached 0 degrees. The Test pianist had five moments of looking forward (around 70 degrees at 5, 65, 220, 265, and 315 s), a moment of looking away from the virtual agent (100 degrees at 170 s) and looking slightly inclined toward the virtual agent for the majority of the performance (around 40 degrees).

Figures 6H–8H shows recurrence plots of the postural sway of the Test pianist in each condition. At first sight, one can see a phase transition in the Human condition at 300 s. This moment showed a decrease in gaze angle and the start of a musically synchronized period. Looking at postural sway, it shows how the Test pianist adapted her posture at that time for the remainder of the performance. In addition, one can see the clusters of rectangular recurrence regions that, given a certain delay, correspond to the different bars relatively well. Recurrence values for the Agent condition are regular but smaller on average compared to other conditions. This finding indicates an even spread of recurrences within each point’s radius or a uniform noise component for the phase space trajectories.

#### Layer 3: Subjective Experience

For the qualitative aspects, we focused on the subjective experience of the Test pianist after the interaction. The analysis goals were to evaluate how the Test pianist experienced each performance globally and in specific moments.

Global scores on the interaction from the custom questions showed satisfactory enjoyment for the Human and Avatar conditions [(HC, AvC, AgC) = (7,7,4)], the most natural interaction in the Human condition [(HC, AvC, AgC) = (4,1,1)] and interestingly, higher scores for experienced closeness to the musical partner in the Avatar condition [(HC, AvC, AgC) = (4,6,1)]. The Test pianist commented about the Avatar condition how “*the VR environment added solely an interesting, fun element that was almost discarded when the actual playing took place. Because I had a good interaction with my partner, the feeling was very close to the one of a performance that happens in real conditions. The fact that the other pianist was responsive to me was enough to convince me that the situation was real and made me enjoy it thoroughly*.” The Agent condition had lowest scores on enjoyment, closeness, and naturalness. Comments of the Test pianist revealed frustration caused by a non-intentional virtual agent: “*It took me about one to two minutes to realize that my virtual partner was, in fact, not present. … My main focus was on trying to understand the intentions of something that was quite obviously not going to follow mine. … as opposed to the second condition, where the fact that I felt the presence of a real person made me connect immediately to an image that was obviously not real, in the third condition I felt almost repulsed by the visual element*.”

**Figure 9:**
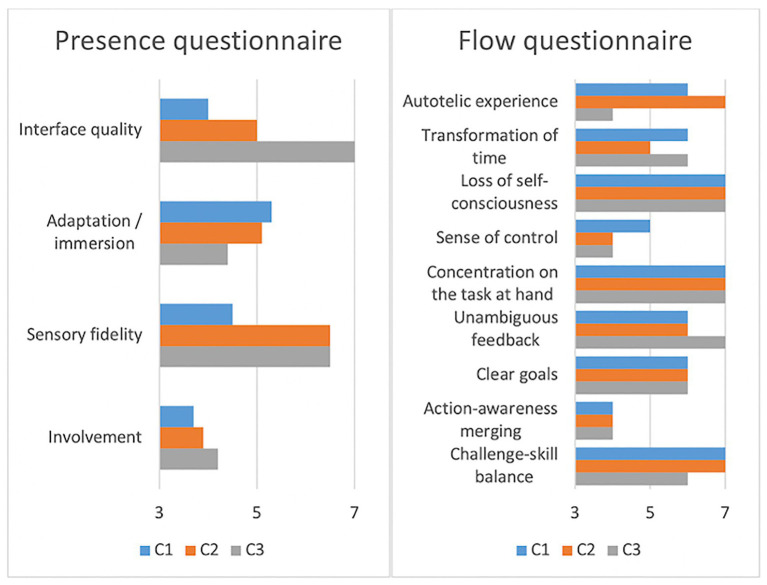
Presence and flow scores of the Test pianist in all conditions (scores ranging from 1 = Strongly disagree to 7 = Strongly agree for the flow questionnaire and from 1 = Not at all to 7 = Completely for the presence questionnaire).

The immersive tendencies questionnaire did not detect anomalies in the Test pianist’s profile (involvement = 6.38, focus = 6.57, games = 5.00). Presence and flow scores are shown in Figure 9. Flow scores were relatively close across conditions (mean_HC_ = 6.00, mean_AvC_ = 5.89, mean_AgC_ = 5.67) with slightly less challenge-skill matching and autotelic experience in the Agent condition. Presence scores were comparable across conditions as well, with the Agent condition having higher scores on interface quality and involvement and lower scores on immersion. Additional questions in the presence questionnaire (Witmer et al., 2005) had comparable scores across conditions for the “sense of being-there” (HC = 6, AvC = 7, AgC = 7), low scores in “spatial presence” for the Avatar condition (HC = 6, AvC = 1, AgC = 7), and low scores in “similarity to a real place” for the Agent condition (HC = 3, AvC = 5, AgC = 1).

Pupil dilatations of the Test pianist are shown in Figures 6I–8I. It remained too challenging to extract meaningful insights or perform comparisons across conditions due to a technical issue in the Human condition (missing data for 2.5 min) and different light conditions across conditions (pass-through camera in the Human condition while a visualization of the virtual environment in the Avatar and Agent conditions). We do report the normalized data in the figures as an example of a quantitative, physiological method for measuring mental activity in our proposed methodological framework.

### Discussion

Creating a shared context is a delicate process and emerges out of the on-going process of participatory sense-making between closely coupled and coordinated individuals (De Jaegher and Di Paolo, 2007). Once established, second person information in the interaction will have characteristics, such as self-directedness, contingency, reciprocity, affective engagement, and shared intentional relations (Moore and Barresi, 2017). This might have taken place in the Avatar condition as it shows an excellent musical performance with close coordination between pianists. Pianists mirror each other’s posture, move, and de-phase together in time, with joint-actions like synchronized head nods at specific moments in the score. It seems as if both pianists have the interpersonal coordination necessary to co-create the musical structure and perform music as a group, anticipating and adapting to each other effectively (D’Ausilio et al., 2015; Walton et al., 2018b). Head movement analyses showed synchronized musicians moving along with the metrical time of the score across conditions. Phase angles between these movements drifted during the performance reflecting the changing relative phase resulting from instructed accelerations. Musicians embodied musical time, keeping time for their own stable or accelerating part in the composition and, given the absence of external timekeeping, created time together (Walton et al., 2018a).

A shared context implies the participants are involved in participatory sense-making (De Jaegher and Di Paolo, 2007) with joint-actions and the presence of intentional relations in subjectivity or consciousness, or a form of intersubjectivity (Zahavi, 2001). Taking the second person approach to social understanding requires an understanding of these intentional relations (Moore and Barresi, 2017). The relevance of virtual scenarios, avatars, and agents for this approach is exemplified by the application of our methodological framework on the Avatar and Agent conditions in our case-study. Self-reports from the Test pianist directly referred to “the presence of a real person” about the avatar in the Avatar condition and the realization about the agent in the Agent condition “that my virtual partner was, in fact, not present” while she was “trying to understand the intentions of something [the agent].” The comments also stressed the difference between interacting (social presence) as opposed to being with another (co-presence) (Garau et al., 2005; Parsons et al., 2017). The other layers in our framework further demonstrate the diffuse intentional relations in the Agent condition by a turbulent coordination and less movement synchronization resulting from a lack of communication between the pianists. As the Test pianist commented about a perceived “non-intentional agent,” it might have been unclear for the Test pianist when the performance was in a (de)synchronizing measure of the score.

While the agent in the Agent condition had natural movements recorded from real performances, the Test pianist did not synchronize as well musically and behaviorally as compared to the other conditions. Nevertheless, musical structure was still reflected in the Test pianist’s body sway corresponding to findings from earlier research that showed the transfer of musical structure in musician’s movements (Demos et al., 2018) and possibly indicating individual time keeping. The virtual agent and musical structure of the Agent condition could have been too rigid for the Test pianist to coordinate effectively, resulting in informationally and behaviorally decoupled musicians. The agent might have lacked adaptive flexibility (Walton et al., 2018b) and the ability to actively distort or co-determine the musical structure (Doffman, 2009; Walton et al., 2018a).

Flow questionnaire scores were comparable across conditions. The presence questionnaire showed a low sense of spatial presence in the Avatar condition possibly resulting from the closer coordination demonstrated by the synchronized head nodding, the gaze angle, and self-reports. Higher scores for involvement and interface quality in the presence questionnaire of the Agent condition might have resulted from the frustration of interacting with the non-intentional agent. It might have made the virtuality of the agent become more obvious and caused the Test pianist to become more (negatively) emotionally involved and become more aware of playing with the piano interface. Musical performance in the Agent condition was not satisfactory for the pianist despite relatively satisfactory relative phase progression and joint RQA metrics. On the other hand, scores indicated a successful execution and enjoyment of the performance in the Human and Avatar conditions.

We argue that the combined successful musical performance, close coupling and shared intentional relations across different layers between the interacting individuals could be necessary and sufficient conditions for the feeling of social presence. Our methodological framework allowed to frame and couple patterns in these dynamics while adhering to the proposed multi-layered notion of presence (Riva et al., 2004) that is “rooted in activity” (Slater et al., 2009). VR has been the core enabler in this framework through its unparalleled flexibility in controlling stimuli and as an approximation of the ideal, nonmediated interactions we have in real life.

## General Discussion and Future Work

The design and analysis of the case-study based on our proposed methodological framework have allowed us to describe performative, behavioral, and experiential interaction dynamics across real and virtual conditions. The comparison of dynamics from the virtual interactions with the real-life setting has provided the means to evaluate similarities and differences that we argue are needed to confirm the required level of social presence for ecologically valid social cognition research. While music interactions represent a particular setting for the study of social cognition, they are able to create shared contexts with object-centered interactions that involve emotional engagement with joint attention and joint goal-direction actions (Moore and Barresi, 2017). As such, their analysis could support the move toward a second person approach to social understanding and help close the gap between first person experiential and third person observational approaches (Schilbach et al., 2013).

A first extension of this study would be moving from a case-study to a full experiment involving a large number of participants. One could then move from the descriptive analyses above to statistically substantiated and quantifiable claims about social presence within specific (music) interaction contexts using the methodological framework introduced in this paper. Recording (neuro)physiological signals in the subjective layer using biosensors or a hyperscanning setup (Dumas et al., 2011) would also help to support these claims.

One could vary aspects of the virtual environment as presence modulators to influence the perceived level of realism. An interesting direct modulator would be to blend virtual and real worlds using augmented reality technology. To gain deeper insights into the constitutive aspects of the feeling of social presence, an interesting variation would be experimenting with different performance settings by varying the environment’s acoustics or the inclusion of an audience. For example, one could evaluate the changes in coordination dynamics and experience of the pianists by processing the audio to simulate a dry practice room or reverberant concert hall. With inclusion of an audience, one could vary its engagement (Glowinski et al., 2015).

Besides controlling the (perceived) realism of the environment, one could also modulate the (perceived) realism of the virtual avatar and agents. One could include some form of emotional content by varying facial expressions, include eye-blinking and gaze, have the agent mirror posture of participants or include behavioral cues at transitional moments of (de)synchronization in the form of head nods. Interaction with the virtual agent might be improved by incorporating elements of surprise and controlled variability. One could script specific actions, blend multiple animations providing richer gesture sets, or leverage machine learning techniques to learn new interaction dynamics that balance the exploration and exploitation of possible behavior states. Specifically, the Kuramoto model used in our case-study could be extended to incorporate richer dynamics and sudden transitions as in the models developed in other research (Mörtl et al., 2014; Shahal et al., 2020; Tognoli et al., 2020). While the main differentiator between conditions has been the behavioral realism of the virtual humans, future studies could vary their appearance realism as well and investigate possible influences on interaction dynamics and social presence (Bailenson et al., 2006; Roth et al., 2016).

Another avenue of investigation would be the inclusion of a multi-user VR scenario. Instead of having one test-subject interacting with a real human, avatar, or agent, one could place both pianists in the virtual environment and analyze the dynamics of both participants individually and together. This would require the combination of detailed finger tracking and full body movement as well as low latency, synchronized, audiovisual, and tactile content. Multi-user VR scenarios can also be designed as networked performances with investigations into aspects of spatial presence. These are technically challenging but feasible using the technology presented in this paper.

Finally, the methodology presented here is readily available to offer better understanding in existing dynamical theories of action and perception (Warren, 2006), social psychology (Tarr et al., 2018) as well as open new research pathways such as VR based music cognition research and support the investigation of subjective qualities prevalent in musical interactions, such as presence, flow, agency, and togetherness (Herrera et al., 2006; Nijs et al., 2012; Shirzadian et al., 2017). The set-up could be used to transfer existing paradigms in joint-action and amnestic re-embodiment (Suzuki et al., 2012; Metzinger, 2018) to musical interaction scenarios as well as for applications in education and creative works. The latter was demonstrated by a public performance in our Art and Science Interaction lab using a modified version of the virtual environment described in this paper.^1^

## Conclusion

We introduced a multi-layered methodological framework incorporating qualitative and quantitative methods to assess the feeling of social presence in social music interactions in virtual reality. We then applied this framework on a case-study involving a duet piano performance in which an expert pianist played a musical composition with another expert pianist; a human-embodied avatar controlled by an expert pianist; and a computer-controlled agent. The case-study showed excellent performances with close interpersonal coordination in behavioral and experiential layers for interactions between the real pianist and virtual avatar and a good performance without interpersonal coordination for the interaction with the virtual agent. The analyses demonstrated the potential of our proposed framework in assessing social presence as well as in highlighting opportunities and challenges in developing better virtual interactions with and models of virtual humans.

## Data Availability Statement

The datasets presented in this study can be found in an online repository. The repository can be found at: https://github.com/ArtScienceLab/Piano-Phase.

## Ethics Statement

The studies involving human participants were reviewed and approved by Ghent University Faculty of Arts and Philosophy Ethics Committee. The participants provided their written informed consent to participate in this study.

## Author Contributions

BK co-designed the study, developed the experimental set-up, conducted experiments, performed data analysis, and co-drafted the manuscript. GC co-designed the study and provided the performance data for creation of the avatar. P-JM co-designed the study and co-drafted, revised, and approved the manuscript. All authors contributed to the article and approved the submitted version.

### Conflict of Interest

The authors declare that the research was conducted in the absence of any commercial or financial relationships that could be construed as a potential conflict of interest.

## Footnotes

^1^A video is available at: https://youtu.be/GlVaMPCotzM.
